# Genotyping and Plasma/Cerebrospinal Fluid Profiling of a Cohort of Frontotemporal Dementia–Amyotrophic Lateral Sclerosis Patients

**DOI:** 10.3390/brainsci11091239

**Published:** 2021-09-19

**Authors:** Mara Bourbouli, George P. Paraskevas, Mihail Rentzos, Lambros Mathioudakis, Vasiliki Zouvelou, Anastasia Bougea, Athanasios Tychalas, Vasilios K. Kimiskidis, Vasilios Constantinides, Spiros Zafeiris, Minas Tzagournissakis, Georgios Papadimas, Georgia Karadima, Georgios Koutsis, Christos Kroupis, Chrisoula Kartanou, Elisabeth Kapaki, Ioannis Zaganas

**Affiliations:** 1 Neurogenetics Laboratory, Neurology Department, Medical School, University of Crete, 71003 Heraklion, Greece; bourbouli.mara@gmail.com (M.B.); mathiouslp@gmail.com (L.M.); spyroszaf@msn.com (S.Z.); tzagourn@med.uoc.gr (M.T.); 2 1st Department of Neurology, School of Medicine, National and Kapodistrian University of Athens, Eginition Hospital, 11528 Athens, Greece; geoprskvs44@gmail.com (G.P.P.); mrentzos@med.uoa.gr (M.R.); vzouvelu@med.uoa.gr (V.Z.); annita139@yahoo.gr (A.B.); vassilis.kon@hotmail.com (V.C.); gkpapad@yahoo.gr (G.P.); gkaradim@med.uoa.gr (G.K.); gkoutsis@med.uoa.gr (G.K.); chrisoulakart@hotmail.com (C.K.); ekapaki@med.uoa.gr (E.K.); 3 2nd Department of Neurology, School of Medicine, National and Kapodistrian University of Athens, Attikon University General Hospital, 12462 Athens, Greece; 4 Department of Neurology, Papageorgiou General Hospital, 56403 Thessaloniki, Greece; atichalas@yahoo.com; 5 1st Department of Neurology, AHEPA Hospital, Aristotle University of Thessaloniki, 54621 Thessaloniki, Greece; kimiskid@auth.gr; 6 Department of Clinical Biochemistry, Attikon University General Hospital, Medical School, National and Kapodistrian University of Athens, 12462 Athens, Greece; ckroupis@med.uoa.gr

**Keywords:** frontotemporal dementia, amyotrophic lateral sclerosis, genetics, biomarkers, *C9orf72*, *TARDBP*, *GRN*, *VCP*

## Abstract

Frontotemporal dementia (FTD) and amyotrophic lateral sclerosis (ALS) are part of the same pathophysiological spectrum and have common genetic and cerebrospinal fluid (CSF) biomarkers. Our aim here was to identify causative gene variants in a cohort of Greek patients with FTD, ALS and FTD-ALS, to measure levels of CSF biomarkers and to investigate genotype-phenotype/CSF biomarker associations. In this cohort of 130 patients (56 FTD, 58 ALS and 16 FTD-ALS), we performed *C9orf72* hexanucleotide repeat expansion analysis, whole exome sequencing and measurement of “classical” (Aβ_42_, total tau and phospho-tau) and novel (TDP-43) CSF biomarkers and plasma progranulin. Through these analyses, we identified 14 patients with *C9orf72* repeat expansion and 11 patients with causative variants in other genes (three in *TARDBP*, three in *GRN*, three in *VCP*, one in *FUS*, one in *SOD1*). In ALS patients, we found that levels of phospho-tau were lower in *C9orf72* repeat expansion and *MAPT* c.855C>T (p.Asp285Asp) carriers compared to non-carriers. Additionally, carriers of rare *C9orf72* and *APP* variants had lower levels of total tau and Aβ_42_, respectively. Plasma progranulin levels were decreased in patients carrying *GRN* pathogenic variants. This study expands the genotypic and phenotypic spectrum of FTD/ALS and offers insights in possible genotypic/CSF biomarker associations.

## 1. Introduction

It has been widely recognized that Frontotemporal Dementia (FTD) and Amyotrophic Lateral Sclerosis (ALS) occupy the two extremes of the same pathophysiological spectrum, sharing several histological features and genetic causes [1]. FTD is a highly inherited disorder, with 30–50% of patients reporting family history of a similar phenotype [2]. For this familial form of FTD, several genes, such as the *C9orf72*, *MAPT, GRN*, *TARDBP* and *VCP* genes, have been found to harbor pathogenic variants [3,4]. On the other hand, in the 10% of patients with the familial form of ALS and in a proportion of the sporadic cases, causative variants have been identified in an increasing number of genes [5]. Interestingly, many patients display both the FTD and the ALS phenotype, often associated with a specific gene variant [6]. Additionally, a pathogenic FTD/ALS gene variant can cause differing phenotypes (FTD, ALS or both FTD and ALS) in different members of the same family [1]. 

Recent studies have shown that variants in the FTD/ALS-associated genes or FTD/ALS-specific neuropathological changes may cause neurodegenerative phenotypes beyond the typical FTD/ALS presentation [7,8,9,10]. Inversely, FTD/ALS clinical features may be associated with non-FTD/ALS pathology [11,12]. These cases are clinically indistinguishable; however, biomarkers, such as those derived from the cerebrospinal fluid (CSF), offer clues to their diagnosis. These CSF biomarkers, namely total-tau protein (**τ**_T_), phosphorylated-tau protein (**τ**_P_) and β-amyloid peptide with 42 amino acids (Aβ_42_) have already been successfully incorporated in Alzheimer’s Disease (AD) diagnostic criteria used for research purposes [13,14]. For FTD-ALS, TDP-43 protein in the CSF is an emerging biomarker [15,16]. However, there is still uncertainty about the interplay between genetic variants and CSF biomarkers.

The aim of the present study was to identify causative variants in the FTD and ALS- associated genes in a well-characterized (including CSF biomarker profiling) cohort of Greek patients presenting with FTD, ALS or FTD-ALS phenotypes. Additionally, we aimed to investigate the association of the genotype with the clinical phenotype and the levels of CSF (**τ**_T_, **τ**_P-181_, Aβ_42_, TDP-43) and plasma (progranulin) biomarkers. 

## 2. Materials and Methods

### 2.1. Participants

A total of 130 patients were included in our study, presenting either to the 1st Department of Neurology of the National and Kapodistrian University of Athens at Eginition Hospital, Athens Greece, the University Hospital of Heraklion, Crete and the Papageorgiou and AHEPA Hospitals, Thessaloniki, Greece. Patients were prospectively enrolled between 2014 and 2019. For inclusion in the study, patients had to receive the diagnosis of FTD, ALS or FTD-ALS according to widely accepted criteria (see below). For the FTD patients, exclusion criterion was the presence of an AD CSF biomarker profile, defined according to the Neurochemistry Unit of the 1st Department of Neurology, University of Athens cutoff values (Aβ_42_ ≤ 580 pg/mL, **τ**_T_ ≥ 376 pg/mL and **τ**_P-181_ ≥ 62.5 pg/mL) [16]. 

The 130 patients were divided into three well-characterized groups:

The FTD group consisted of 56 patients who met the criteria for either the behavioral subtype of FTD (bvFTD) [17] or primary progressive aphasia (PPA), regardless of the subtype [18]. Of the PPA patients who eventually participated in the study, 6 met the semantic variant PPA (svPPA) criteria and 5 met the non-fluent agrammatic PPA (nfaPPA) criteria. 

The ALS group included 58 patients who met the Awaji-Shima criteria [19]. 

The ALS-FTD group included 16 patients who met the criteria of the combined ALS-FTD phenotype [20].

All patients underwent detailed clinical, neuropsychological, biochemical and neuroimaging examination (magnetic resonance imaging [MRI] in all patients and, additionally, single-photon emission computed tomography [SPECT] in most FTD patients) to exclude secondary causes of dementia and to establish the diagnosis of FTD, ALS, or FTD/ALS.

The study was performed according to the ethical guidelines of the 1964 Declaration of Helsinki and had the approval of the Scientific and Ethics Committee of all hospitals involved. Informed consent was obtained from each subject when possible or their authorized caregiver(s).

### 2.2. Blood Collection and DNA Extraction

For the genetic analyses, whole peripheral blood from participants was collected in ethylenediaminetetraacetic acid (EDTA) tubes, which were stored at −20 °C until DNA extraction and were only thawed once, just before the procedure. For the extraction of the genomic DNA from 400 μL of whole peripheral blood, the QIAamp DNA Blood Mini kit (Qiagen, CA, USA) was used. DNA concentration and purity were assessed spectrophotometrically at 260 and 280 nm. Participant anonymity was ensured through the designation of unique code identifiers for each DNA sample.

### 2.3. C9orf72 Repeat Expansions

Most of the patients (*n* = 94) were initially checked for *C9orf72* hexanucleotide repeat expansions at the Neurogenetics Laboratory of Eginition Hospital according to a two-step protocol. This protocol included sizing PCR (amplification of the region that contains the hexanucleotide repeat with primers flanking this region), agarose electrophoresis and fragment analysis to identify samples with possible presence of the *C9orf72* repeat expansion. This was followed by repeat primed PCR, to verify and separate samples with the *C9orf72* repeat expansion, as previously described [21,22]. Samples were then electrophorized on an ABI 310 Capillary Analyzer (Applied Biosystems, Foster City, CA, USA) and analyzed on GeneScan v3.7 (2001, Applied Biosystems, Foster City, CA, USA). In addition, 21 patients were initially analyzed for the presence of *C9orf72* repeat expansion by repeat primed PCR amplification and STR (short tandem repeats) PCR analysis at the Diagnostic Service Facility, Laboratory of Neurogenetics, University of Antwerp, Belgium.

### 2.4. Whole Exome Sequencing

**Figure 1:**
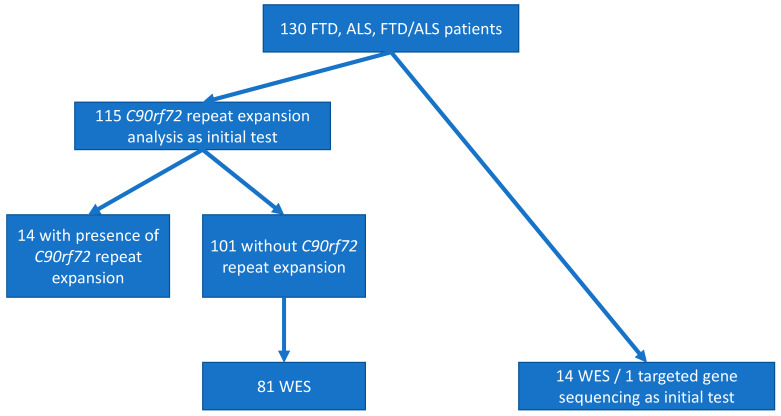
Flow chart of the genetic characterization of FTD and/or ALS patients in the cohort we analyzed. Most (115 out of the 130) patients were analyzed by *C9orf72* repeat expansion analysis as the initial test. Subsequently, 81 of the 101 *C9orf72* repeat expansion negative patients were analyzed by WES (whole exome sequencing). For 14 patients, WES was chosen as the initial test, and for 1 (with a pathogenic variant in the *VCP* gene), targeted gene sequencing was performed initially due to clinical picture and family history highly suggestive of inclusion body myopathy, Paget’s disease and FTD [23].

In most patients that did not carry a *C9orf72* hexanucleotide repeat expansion, Whole Exome Sequencing (WES) was performed (Figure 1) according to the following procedures:

(1) At Minotech Genomics Facility, Institute of Molecular Biology and Biotechnology (IMBB-FORTH, Crete) with the use of the Illumina NextSeq500 platform (*n* = 60). In detail, sequencing of 2 × 75 bp DNA fragments with at least 50× coverage, targeting regions of 45.3 Mb size, was performed. Libraries were prepared with the TruSeq^®^ Rapid Exome Library prep kit (Illumina, San Diego, CA, USA). Bioinformatics processing of the data derived from mapping on the hg19 reference genome and quality control of the results (e.g., number of readings and coverage quality) were performed by the BaseSpace^®^ software (Illumina, San Diego, CA, USA). Finally, genetic variation was identified with the VariantStudio^®^ software after comparison with the hg19 reference genome and drawing information from genetic databases, e.g., Human Genome Mutation Database (HGMD), ClinVar^®^ (National Center for Biotechnology Information, Bethesda MD, USA) (Annotation Excel file).
(2) At Macrogen (Seoul, Korea), using the Illumina HiSeq4000 platform (*n* = 24). In specific, 2 × 100 bp DNA fragments were sequenced with an aim of at least 50x coverage. For the construction of genomic libraries, the Agilent Sure-Select Human All Exon V5 (not including UTRs) Target Enrichment System was used.
(3) At Otogenetics (GA, USA), using the Illumina HiSeq2500 platform (*n* = 11). In detail, sequencing of 2 × 100 bp DNA fragments was performed aiming at coverage of at least 50× and targeting a region of 45.3 Mb, that represents >98% of the human coding sequence according to the Consensus Coding Sequences (CCDS) and Ensembl. Exon-enriched library preparation was performed with the use of the Agilent V5 (51Mb) Sure-Select Target Enrichment System.

### 2.5. Gene Variant Identification and Verification

We specifically searched for variants in the most recognized FTD and ALS related genes, namely *C9orf72*, *GRN*, *MAPT*, *TARDBP, FUS*, *CHMP2B, SQSTM1*, *UBQLN2, VCP, OPTN, TBK1, SOD1* and *CHCHD10*, as well as the 3 genes associated with autosomal dominant AD (*APP*, *PSEN1*, *PSEN2*). Single nucleotide polymorphism (SNP) variants reported here were verified by Sanger sequencing to exclude false positive results. 

### 2.6. Measurements of CSF and Plasma Biomarkers

The CSF levels of Aβ_42_, **τ**_T_ and **τ**_P-181_ were measured in duplicate by commercially available ELISA kits (Innotest β-amyloid 1–42, hTau antigen and phospho-tau 181; Fujirebio, Gent, Belgium) according to the manufacturer’s instructions. We chose to measure **τ**_P-181_, as in neurodegenerative disorder research and clinical practice, tau phosphorylated at threonine 181 (**τ**_P-181_) is the most commonly measured form of phosphorylated tau as a biomarker in the CSF [24] and, recently, in plasma [25]. Additionally, to better evaluate the **τ**_P-181_ levels, the ratio of **τ**_P-181_ to **τ**_T_ (**τ**_P-181_/**τ**_T_) was calculated [26]. TDP-43 was measured in triplicate by double-sandwich enzyme-linked immunosorbent assay (ELISA) using a commercial kit (Human TAR DNA-binding protein 43 ELISA kit; Cusabio Biotech, Wuhan, China). All determinations were performed using a four-parameter logistic curve and blindly to the clinical diagnosis. Cut-off levels were calculated by ROC (receiver operating characteristic) analysis with the optimal combination of sensitivity and specificity, as previously described [15,16]. 

Blood samples were collected between 08:00–10:00 a.m. (morning samples), transferred to EDTA-containing tubes and refrigerated until centrifugation (within 3 h) for the plasma isolation. Plasma isolated from these samples was subsequently kept in deep freeze (−80 °C). Plasma progranulin levels were measured using the Human Progranulin ELISA Kit (Adipogen Life Sciences, Liestal, Switzerland) [27]. 

### 2.7. Statistical Analysis

All numerical data were tested for normality and homogeneity of variances by the Shapiro–Wilk’s and Brown–Forsyth tests, respectively. When appropriate, differences among groups were tested by one-way analysis of variance (one-way ANOVA) or two-way analysis of covariance (two-way ANCOVA), followed by Bonferroni correction for multiple comparisons. When deviations from normality and/or heterogeneity of variances were noted, Kruskal–Wallis test was performed, followed by Dunn’s post-hoc test. Categorical data were compared among groups by the χ^2^-test.

## 3. Results

### 3.1. Demographic Data

Demographic data are summarized in Table 1. The three patient groups (FTD, ALS, FTD-ALS) had comparable age and sex. Due to violations regarding normality and homogeneity of variances, disease duration was initially compared among groups with Kruskal–Wallis test that showed a significant difference (*p* < 0.0001). Additionally, Dunn’s post-hoc test revealed significantly longer disease duration for FTD as compared to ALS patients (*p* < 0.0001). 

### 3.2. Family History

Among the 130 patients within the FTD-ALS spectrum, 28.6% (16/56) of the FTD patients had at least one first degree relative affected with phenotype suggesting either FTD or ALS. For the ALS and FTD-ALS groups, 15.5% (9/58) and 25.0% (4/16), respectively, had positive family history for possible FTD or ALS, with at least one first degree relative affected (Table 1). 

### 3.3. Pathogenic and Likely Pathogenic Variants in FTD-ALS Genes 

#### 3.3.1. *C9orf72* Repeat Expansion

The percentage of patients positive for *C9orf72* repeat expansion (defined as presence of >30 repeats) was 10.4% in patients with FTD (5 out of 48 patients tested), 10.7% in patients with ALS (6/56) and 27.3% in patients with FTD-ALS (3/11). All *C9orf72* repeat expansion positive patients in the FTD-ALS group had family history of possible FTD/ALS and presented with a bvFTD phenotype at onset. The clinical and other characteristics of the 14 patients harboring a *C9orf72* repeat expansion are shown in Table 2. 

#### 3.3.2. Other Causative Variants

From the analysis of the FTD/ALS genes in the WES derived data, causative variants were identified in the *TARDBP*, *GRN*, *VCP*, *SOD1* and *FUS* genes in **11** patients (Table 3). Specifically, in three patients (two with pure ALS and one with FTD-ALS) we found causative variants in the *TARDBP* gene, namely the p.Met337Val (c.1009A>G), the p.Asn352Ser (c.1055A>G) and the p.Ile383Val (c.1147A>G) variant.

In three patients with FTD, all with the PPA phenotype, we found three causative variants in the *GRN* gene (Table 3). The two patients with splice site *GRN* variants (c.463-2A>G and c.934-1G>A) presented with an svPPA and nfaPPA phenotype, respectively. Both had increased CSF **τ**_T_ and TDP-43 levels and **τ**_P-181_ and Aβ_42_ levels within the reference range for healthy controls. Another FTD-PPA patient was detected with the p.Cys482Tyr *GRN* variant. She had increased CSF levels of **τ**_T_ and **τ**_P-181_, while the CSF levels of TDP-43 and Aβ_42_ were within the reference range for healthy controls.

Additionally, in three patients with FTD and Inclusion Body Myopathy (IBM), all from Crete, we found two causative variants in the *VCP* gene (Table 3). A 63-year-old male patient with the pathogenic p.Arg159His (c.476G>A) heterozygous missense *VCP* gene variant was identified with the characteristic clinical picture of ΙΒΜ and FTD [23]. The same *VCP* variant (p.Arg159His) was found in a 68-year-old patient with progressive muscle weakness and atrophies in all four extremities and the trunk and behavioral disturbances. The symptoms of the patient had started at the age of 58 years. Another patient was found to have myopathy, persistently increased Creatine PhosphoKinase (CPK), language deficits and Paget’s disease of bone. He carried the p.Arg155His (c.464G>A) *VCP* change (Table 3).

Further, we found ALS causative variants in the *FUS* and the *SOD1* genes (Table 3). Specifically, we detected the p.Gly506Val (c.1517G>T) *FUS* gene variant in a 82-year-old male with a predominately lower motor neuron involvement and no family history. Moreover, we found the p.Ser106Leu (c.317C>T) *SOD1* gene variant in a 37-year old patient with personal and family history of ALS. 

### 3.4. Association of Variants in FTD -ALS Genes with CSF Biomarkers

As a next step in our analyses, we proceeded in measuring CSF biomarker levels in this patient cohort and associated these levels with the results of *C9orf72* repeat expansion determination and WES based genotyping. Our efforts focused on typical neurodegenerative disease-associated CSF biomarkers (TDP-43, Aβ_42_, **τ**_P-181_ and **τ**_T_) and on genes that either have as their protein product the above biomarkers (*TARDBP*, *APP*, *MAPT*) or are commonly associated with FTD/ALS (*C9orf72*). Of these four genes, no genotype/CSF biomarker associations were possible for the TARDBP gene since very few patients with variants in this gene were found.

#### 3.4.1. Association of *C9orf72* Variants with CSF Biomarkers 

Regarding the *C9orf72* gene, in the cohort analyzed, besides the pathogenic hexanucleotide repeat expansion (see above), we found five rare intronic variants (c.444+31T>G, c.600+86A>T, c.666-120C>T, c.855+52A>G, c.856-37G>T) and the p.Asn207Ser (p.N207S) exonic variant. These *C9orf72* variants differ in their location and nature (repeat expansion, intronic and exonic) and thus possibly have differential effects on gene expression and function. For this reason, these three types of genetic variants were analyzed separately. Thus, for this part of our study, we compared Aβ_42_, **τ**_T_, **τ**_p-181_ and TDP-43 CSF levels and the **τ**_P-181_/**τ**_T_ ratio in FTD and/or ALS patients carrying the hexanucleotide *C9orf72* repeat expansion, rare intronic *C9orf72* variants or the p.Asn207Ser (p.N207S) exonic *C9orf72* variant to patients without these variants. Analyses were performed separately for FTD patients, ALS patients ± FTD and all patients combined. 

**Figure 2:**
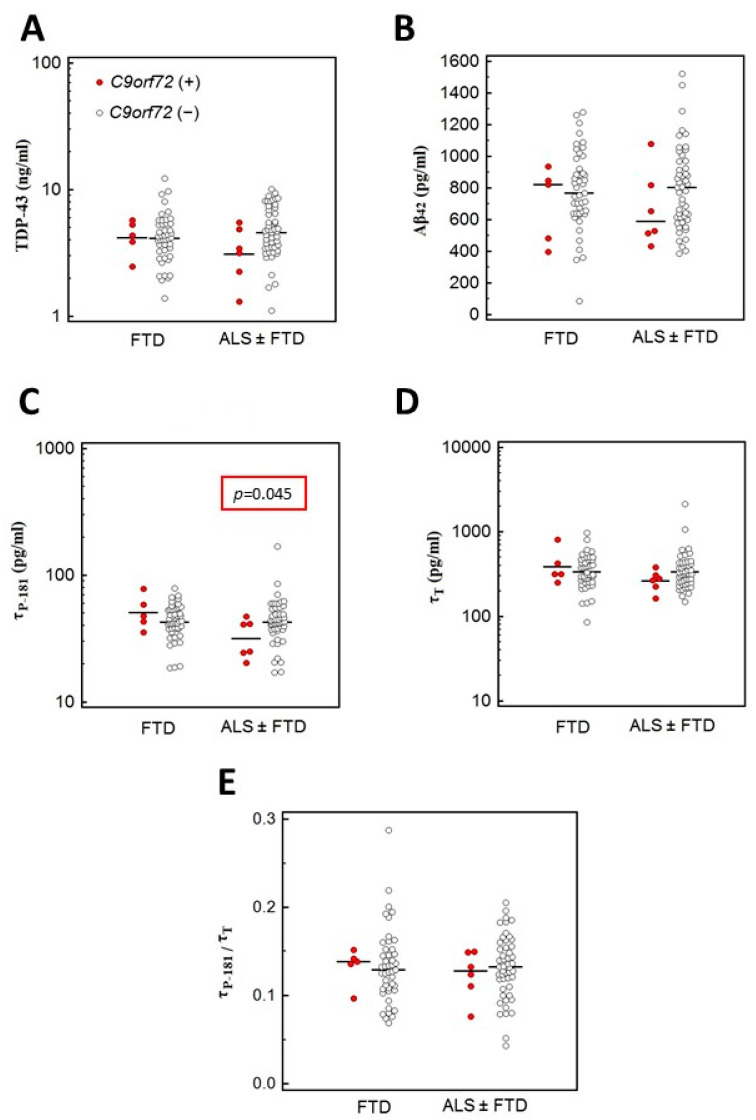
Comparison of TDP-43 (**A**); Aβ_-42_ (**B**); **τ**_P-181_ (**C**) and **τ**_T_ (**D**) levels and **τ**_P-181_/**τ**_T_ ratio (**E**) in *C9orf72* hexanucleotide repeat expansion carriers (*C9orf72*+) and non-carriers (*C9orf72*−). Analyses were performed separately for FTD patients and ALS (with or without FTD) patients. In patients with ALS (with or without concurrent FTD), levels of **τ**_P-181_ were lower in *C9orf72* carriers compared to non-carriers (**C**). All other comparisons yielded non-significant results.

**Figure 3:**
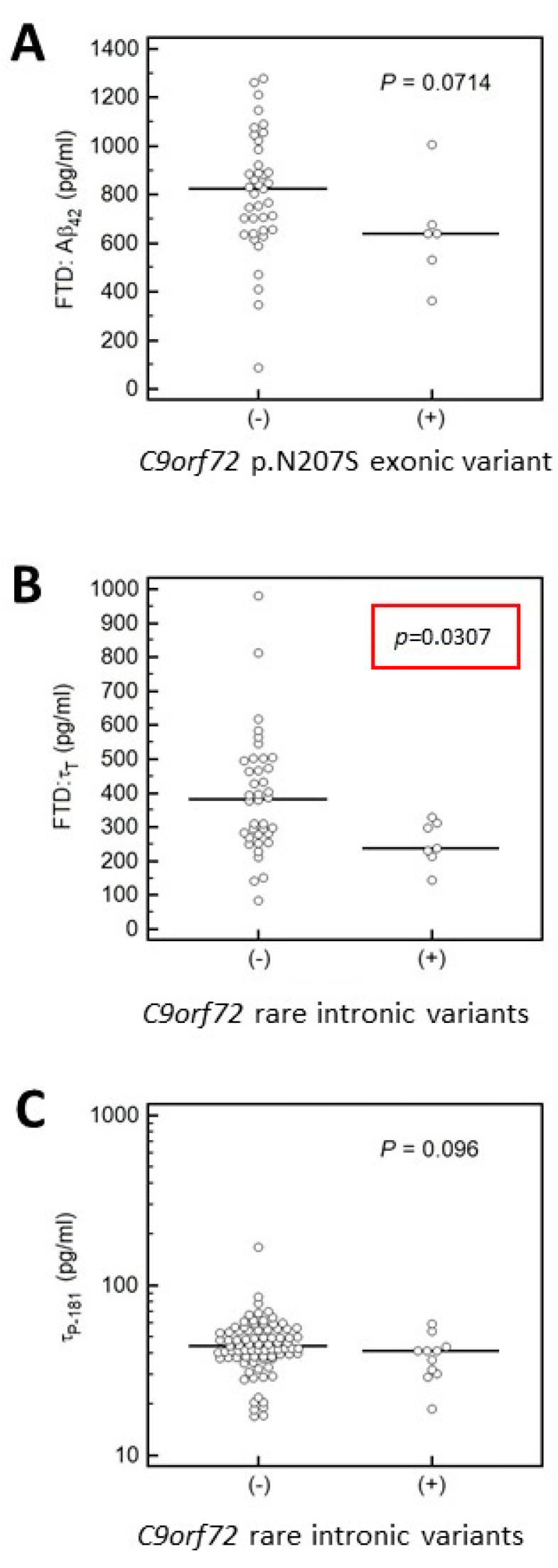
Aβ_42_, **τ**_T_ and **τ**_p-181_ CSF levels in FTD, ALS and FTD/ALS patients carrying either the p.Asn207Ser (p.N207S) exonic *C9orf72* variant (**A**) or the rare intronic *C9orf72* variants (**B**,**C**). (**A**) Here we compared Aβ_42_ CSF levels in FTD patients carrying the p.Asn207Ser (p.N207S) exonic *C9orf72* variant to patients without this variant and no statistically significant difference was found. (**B**) FTD patients that were carriers of rare intronic variants in the *C9orf72* gene had lower CSF levels of **τ**_T_. (**C**) CSF levels of **τ**_p-181_ did not differ in FTD and/or ALS patients carrying rare *C9orf72* intronic variants.

These analyses revealed that in patients with ALS (with or without concurrent FTD), the CSF levels of **τ**_P-181_ were lower in *C9orf72* repeat expansion carriers compared to non-carriers, whereas levels of TDP-43, Aβ_42_ and **τ**_T_ and the **τ**_P-181_/**τ**_T_ ratio did not differ between the two groups (Figure 2). Additionally, carriers of rare intronic variants in *C9orf72* had lower CSF levels of **τ**_T_ (Figure 3). In contrast, this difference in **τ**_T_ levels was not found for the more common *C9orf72* p.Asn207Ser (p.N207S) variant (Figure 3). Results on *C9orf72*-based biomarker level comparisons that did not reveal statistically significant results or results not approaching statistical significance are not shown. 

#### 3.4.2. Association of *MAPT* and *APP* Gene Variants with CSF Biomarkers

**Figure 4:**
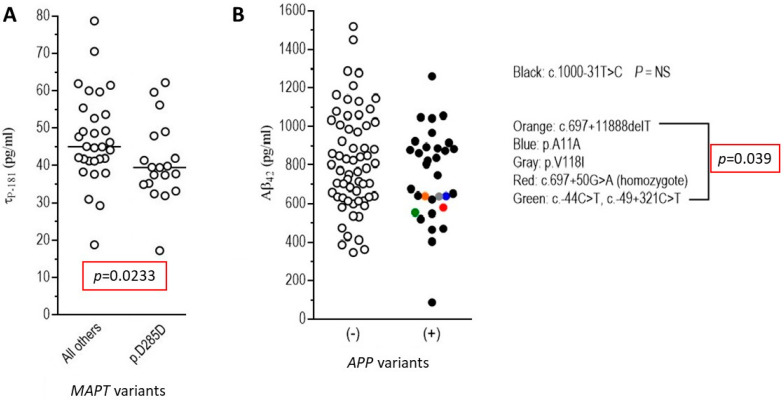
Association of *MAPT* and APP gene variants with CSF biomarkers (Aβ_42_, **τ**_T_, **τ**_p-181_, TDP-43) in the entire cohort of FTD and/or ALS patients. (**A**) As shown here, the c.855C>T (p.Asp285Asp/p.D285D) *MAPT* synonymous variant carriers had significantly lower τ_p-181_ levels compared to non-carriers; (**B**) carriers of rare *APP* variants (p.Ala11Ala/p.A11A, p.Val118Ile/p.V118I, c.-44C>T/c.-49+321C>T, c.697+50G>A and c.697+11888delT) had lower Aβ_42_ levels. These lower Aβ_42_ levels were not found for the more common intronic variant c.1000-31T>C.

When we compared levels of CSF biomarkers between groups of patients carrying different *MAPT* and *APP* gene variants, we found that the c.855C>T (p.Asp285Asp/p.D285D) synonymous *MAPT* variant carriers had significantly lower **τ**_p-181_ (Figure 4). There was no difference found for the other three biomarkers tested (TDP-43, Aβ_42_ and **τ**_T_), when comparing carriers of the p.Asp285Asp (p.D285D) change with non-carriers (data not shown). Additionally, all other *MAPT* variants similarly tested did not show an effect on biomarker levels (data not shown). Additionally, as shown in Figure 4**,** carriers of rare *APP* variants (p.Ala11Ala, p.Val118Ile, c.-44C>T/c.-49+321C>T, c.697+50G>A and c.697+11888delT) had lower Aβ_42_ levels, whereas this was not found for the more common c.1000-31T>C intronic *APP* variant. Finally, other biomarkers (**τ**_T_, **τ**_p-181_, TDP-43) analyzed relative to *APP* variants did not yield significant results (data not shown).

#### 3.4.3. Correlation of CSF Biomarkers Values

**Figure 5:**
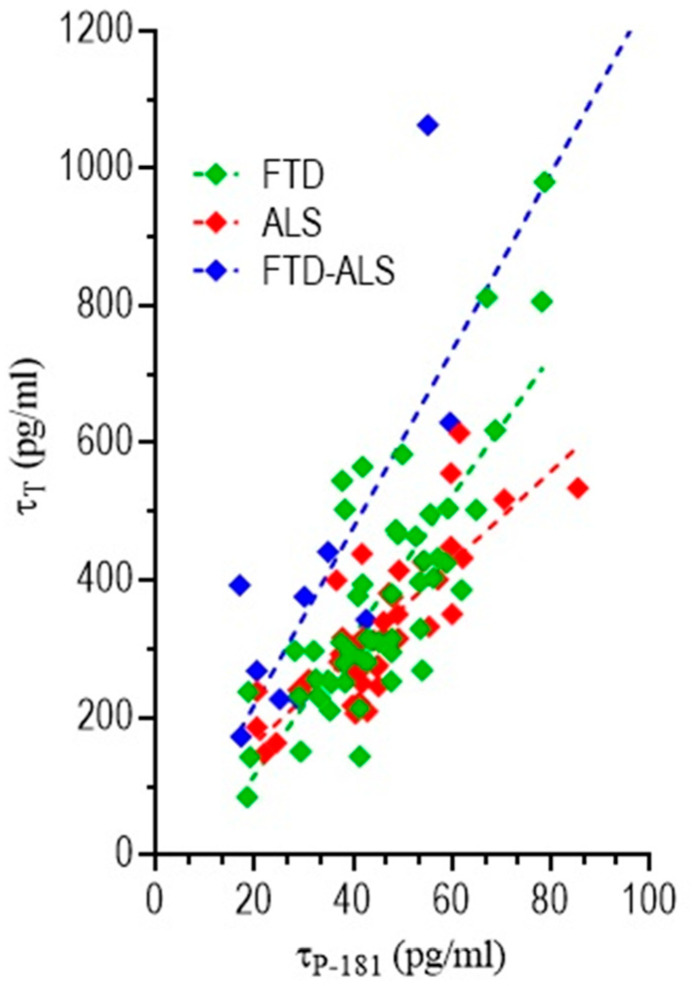
Correlation of **τ**_T_ and **τ**_p-181_ CSF values in different patient groups. Results of these analyses showed that **τ**_T_ and **τ**_p-181_ CSF values were correlated, across all patient groups, with Spearman *ρ* values being 0.74 (*p* < 0.0001) for FTD patients, 0.78 (*p* < 0.0001) for ALS patients and 0.62 (*p* = 0.031) for FTD/ALS patients.

As a next step in our analyses, we performed correlation analyses of CSF biomarker values (Aβ_42_, **τ**_T_, **τ**_p-181_, TDP-43) in pairs across different patient groups. Results of these analyses showed that only **τ**_T_ and **τ**_p-181_ CSF values were correlated, across all three patient groups (Figure 5). For these analyses, Spearman *ρ* values were 0.74 (*p* < 0.0001) for FTD patients, 0.78 (*p* < 0.0001) for ALS patients and 0.62 (*p* = 0.031) for FTD/ALS patients. Correlation between other CSF biomarker measurements pairs (**τ**_P-181_ vs. Aβ_42_, Aβ_42_ vs. TDP-43 and TDP-43 vs. **τ**_P-181_) did not yield statistically significant results (data not shown).

### 3.5. Association of Pathogenic Variants in the GRN Gene with Plasma Progranulin Levels

**Figure 6:**
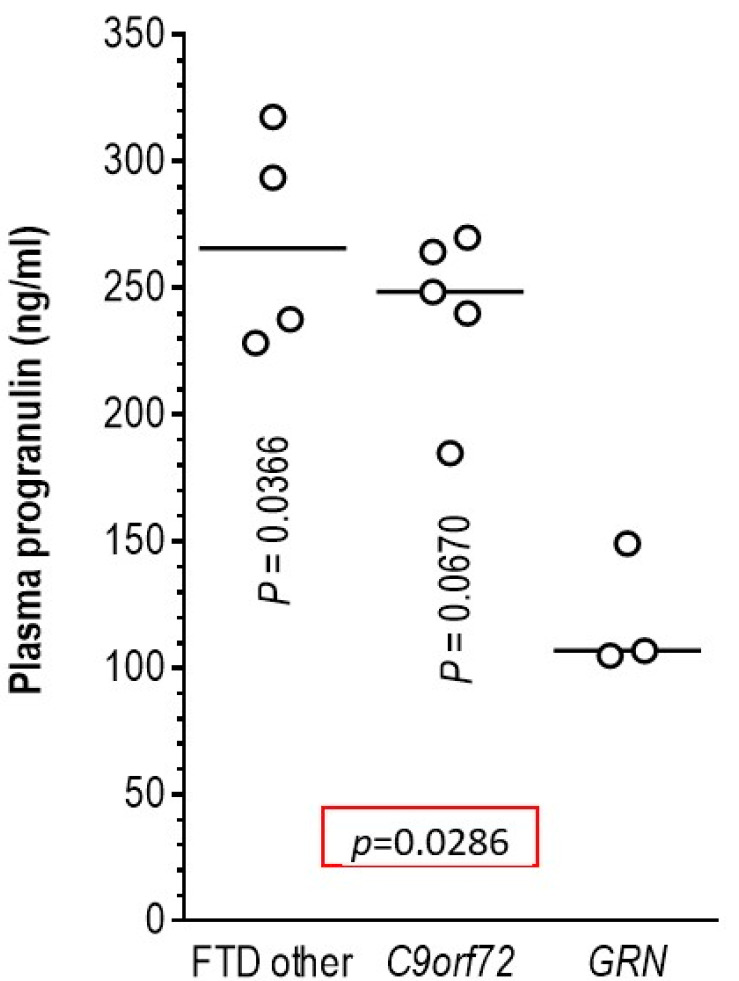
Association of *GRN* pathogenic variants with plasma progranulin levels. Lower plasma progranulin levels were found in the 3 FTD patients with the *GRN* variants (c.463-2A>G, c.934-1G>A and p.Cys482Tyr) compared to FTD patients harboring the *C9orf72* repeat expansion and to other FTD patients.

To show that our findings for CSF biomarkers (i.e., that genetic variants affect CSF biomarker levels) are valid also for plasma biomarkers, we performed plasma progranulin quantification in the three patients with the *GRN* variants, c.463-2A>G, c.934-1G>A and p.Cys482Tyr (c.1445G>A), that were considered causative based on strong in silico predictions. Patients carrying the pathogenic *GRN* variants had more than two-fold lower plasma progranulin levels compared to FTD patients harboring the *C9orf72* repeat expansion and to other FTD patients (Figure 6). These findings verified the pathogenicity of the three novel *GRN* gene variants. 

## 4. Discussion

Our study presents a clinical series of Greek FTD/ALS spectrum patients by innovatively integrating phenotypic, genotypic and biomarker data. Causative genetic variants (either SNPs or *C9orf72* hexanucleotide repeat expansions) were identified in 25 cases (19.2% of the total patient cohort). The frequency of patients carrying causative FTD/ALS gene variants in our series is higher compared to other similar series from Greece, most of them having evaluated either FTD or ALS cohorts separately [28,29,30]. This increased frequency probably reflects a higher proportion of FTD/ALS patients in our study, as well as the exclusion of patients with probable AD pathology through CSF biomarker measurements. Additionally, we have observed several unique associations between genotype and CSF/plasma biomarker levels, with our study being one of the first studies to adopt such an approach.

The most common genetic change detected in the cohort analyzed was, as expected, the *C9orf72* repeat expansion (12.2%; 14 out of 115 patients tested). *C9orf72* repeat expansions have been reported to 4–40% of patients with ALS [31]. In previously reported Greek ALS cohorts, 5–10% of cases carried the abnormal *C9orf72* expansion [28,32], in accordance with our findings. Regarding the FTD group, we found a rate of *C9orf72* repeat expansions (10.4%), similar to one of the studies from Greece [29], but almost two-fold higher compared to another Greek study [30]. The latter can be explained by the biomarker supported clinical diagnosis of FTD patients in our study, avoiding the contamination of this group with other pathologies presenting as a frontal-behavioral or language syndrome. 

Additionally, using a WES based approach, we identified causative gene variants in **11** more patients: three variants in each of the *TARDBP* and *GRN* genes, two variants in the *VCP* gene (in three patients) and one variant in each of the *SOD1* and *FUS* genes (Table 3). Variants in these genes are commonly described in FTD/ALS cohorts, including the most recent studies [33]. 

In detail, we detected three patients (two women with ALS and one man with FTD-ALS) with *TARDBP* pathogenic variants (p.Met337Val, p.Asn352Ser and p.Ile383Val). This is of special importance as *TARDBP* encodes TDP-43, a protein which is increasingly implicated in neurodegenerative processes and offers an attractive therapeutic target [34]. Pathogenic *TARDBP* gene variants are considered the cause of 1–7% of familial ALS cases and of several sporadic ALS cases [35,36], but they are less frequently reported in FTD [3,37]. Our 60-year old patient with the p.Ile383Val *TARDBP* gene variant presented with an FTD-ALS phenotype. This specific variant has been repeatedly described as pathogenic across the FTD/ALS spectrum [33,38,39,40,41]. Additionally, three FTD patients of Greek origin harboring the p.Ile383Val *TARDBP* gene variant have been recently reported [30]. These data are a call for a systematic study of this variant’s geographic distribution and significance. Furthermore, the c.1009A>G (p.Met337Val) *TARDBP* variant found in one of our patients with familial ALS has been repeatedly reported to segregate with ALS [42,43,44,45,46], including bulbar onset ALS [47]. The p.Asn352Ser *TARDBP* variant that we found in another ALS patient has also been often described in the literature [48,49,50] and functionally characterized [51,52].

In three of our patients, we found causative variants in the *GRN* gene (c.463-2A>G, c.934-1G>A and p.Cys482Tyr), all novel (not previously reported) but with strong evidence in favor of their pathogenicity. Variants in the *GRN* gene have been emerging as a frequent cause of FTD, with marked phenotypic heterogeneity [53,54,55] but most frequently associated with TDP-43 histopathology and PPA phenotype [56,57,58]. Our data add further support to the association of *GRN* variants with the PPA phenotype. The two splice site variants (c.463-2A>G, c.934-1G>A) described here have strong in silico evidence that they disrupt splicing, as indicated by their MaxEntScan and CADD scores (Table 3). Additionally, in the *GRN* gene, this type of variant (splice-site variants) is frequently described as causing FTD. In another FTD-PPA patient, we detected the p.Cys482Tyr *GRN* variant, which is essentially absent from public variant databases such as gnomAD and has strong computational evidence (CADD score = 32.0) in favor of its pathogenicity (Table 3). All three patients in the cohort we analyzed harboring causative *GRN* variants showed decreased plasma progranulin levels (Figure 6). This is additional evidence of the pathogenicity of these variants, as it has been shown for other *GRN* variants [27,59].

Three male patients, all originating from Crete and all presenting with the IBM/FTD phenotype, were found to harbor heterozygous pathogenic *VCP* variants. These *VCP* variants were the p.Arg155His (c.464G>A) and, in two apparently unrelated patients, the p.Arg159His (c.476G>A) variant. Pathogenic *VCP* gene variants are associated with the characteristic clinical picture of IBM, FTD and ALS, often with Paget’s disease of bone. This syndrome can be partially or fully developed depending on the respective causative *VCP* variant and other factors [60,61]. The p.Arg155His *VCP* variant has been repeatedly shown to co-segregate with the disease phenotype in multiple affected family members [61,62,63]. This variant is in a mutational hot spot and there are in vitro and in vivo functional studies and strong computational evidence (CADD = 24.6) supportive of its damaging effect. This p.Arg155His *VCP* missense change occurs at an amino acid residue where different missense changes (p.Arg155Pro, p.Arg155Leu, p.Arg155Cys, p.Arg155Ser) are known to be pathogenic [63]. Regarding the p.Arg159His variant, there are several reports describing patients with this variant in the literature [63], including the clinical description of one of our patients [23]. This variant is not present in population databases such as gnomAD, it has strong in silico evidence of pathogenicity (CADD = 23.2) and affects a conserved arginine residue (Table 3). This amino acid change has been shown to disrupt the function and structure of *VCP* and can lead to protein aggregates [64,65,66,67].

In addition, we detected the p.Gly506Val (c.1517G>T) *FUS* gene variant in a patient with ALS. The FUS protein is a widely expressed DNA/RNA-binding protein with functional homology to TDP-43 [68]. It is involved in transcriptional and translational regulation and in mRNA splicing and transport [69]. *FUS* gene pathogenic variants cause 5% of familial and 1% of sporadic ALS, with predominant lower motor involvement [68,70,71]. The p.Gly506Val *FUS* gene variant is not present in public databases, such as gnomAD, and there is computational evidence in support of its pathogenicity (CADD = 23.7). Additionally, there are reports on a different amino acid substitution (as p.Gly507Asp) at the same amino acid residue causing ALS [68,70]. Finally, in a 37-year old male with family history of ALS, we identified the p.Ser106Leu *SOD1* variant. This variant has already been described in young ALS patients with rather slow disease progression, as was the case in our patient [72,73,74].

There have been several studies on the CSF levels of TDP-43, Aβ_42_, **τ**_T_, **τ**_P-181_ in patients with FTD and ALS [15,75], but very few have focused on the association of these levels with the genotype of these patients. Here we found several interesting genotype/CSF biomarker associations. Specifically, we found that levels of **τ**_p-181_ were lower in *C9orf72* repeat expansion and *MAPT* c.855C>T (p.Asp285Asp) carriers compared to non-carriers. Additionally, carriers of rare *C9orf72* and *APP* variants had lower levels of **τ**_T_ and Aβ_42_, respectively. 

Regarding *C9orf72*, it has been reported that 25% of patients with *C9orf72* repeat expansion show low Aβ_42_ levels in the CSF [76], but the effect of this expansion on tau and phospho-tau levels is not clear [77]. Our finding that *C9orf72* changes (either the pathogenic repeat expansion or the rare intronic variants) can affect levels of **τ**_P-181_ and **τ**_T_, indicates that there is an association between *C9orf72* and tau species that needs further assessment. 

The effect of the *MAPT* c.855C>T (p.Asp285Asp) change on levels of **τ**_p-181_ in the FTD/ALS cohort we studied suggests that this variant could be an indirect modifier of tau phosphorylation (e.g., by affecting mRNA processing) and thus of pathophysiological significance. This is of interest, since the same effect, increased levels of **τ**_p-181_, has been found for several *MAPT* variants in patients with AD [78]. Our findings extend the observations about this effect on another neurodegenerative disorder beyond AD.

Finally, the effect of rare *APP* variants on Aβ_42_ levels suggests these variants are possibly important for APP processing by secretases, as it has already been shown for known pathogenic and non-pathogenic *APP* variants in AD and other neurodegenerative disorders [79,80,81].

### Strengths and Limitations

The main strength of our work relates to the extent and depth of the genotypic and phenotypic analysis, that included both WES-based genotyping and CSF/plasma biomarker measurements. 

One limitation to our work is the rather small cohort size; however, this is counterbalanced by the depth of the genotypic and phenotypic characterization. In addition to the plasma and CSF biomarkers used in our study, there is increasing interest in the possible role of other emerging biomarkers in ALS and FTD, including neurofilaments both in CSF [82,83] and plasma [84,85]. This is of special importance for patients with the FTD/ALS phenotype, given that there is evidence that elevated Neurofilament Heavy chain (NfH) levels could antedate the onset of ALS in FTD patients [83]. Our study was not designed to test the levels of these biomarkers in the plasma and CSF; however, future analyses are panned to include these important biomarkers.

## 5. Conclusions

In conclusion, we found causative genetic variants (in the *C9orf72*, *TARDBP*, *GRN*, *VCP*, *FUS* and *SOD1* genes) with a high frequency (19.2%) in a cohort of Greek FTD/ALS spectrum patients. In addition, we observed several potentially important associations between the plasma/CSF biomarker levels and the genotype of several genes. Specifically, we found lower levels of **τ**_p-181_ in *C9orf72* repeat expansion and *MAPT* c.855C>T (p.Asp285Asp) carriers compared to non-carriers. Additionally, carriers of rare *C9orf72* and *APP* variants had lower levels of **τ**_T_ and Aβ_42_, respectively. Finally, plasma progranulin levels were decreased in patients with the newly described pathogenic *GRN* variants. 

Our results further expand the genotypic/phenotypic spectrum of FTD/ALS and provide insights in the interaction of the genome with plasma and CSF biomarkers. Taken together, our findings call for an integrated individualized approach in interpreting plasma and CSF biomarker levels, as these can be influenced by the genotype of the patient under study.

## Figures and Tables

**Table 1:** Demographic and clinical data of the studied groups.

	FTD	ALS	FTD-ALS	*p*-Value
*n* (m/f)	56 (32/24)	58 (26/32)	16 (7/9)	NS ^†^
Age (y)	60.2 ± 10.8	61.2 ± 11.8	60.7 ± 10.7	NS ^‡^
Disease Duration (y)	3.0 (1.3–6.0) *^a^*	1.0 (0.7–2.0)	3.0 (1.0–4.0)	<0.001 ^§^
Family History, 1st degree relative (%)	16 (28.6)	9 (15.5)	4 (25.0)	NS ^†^

Data are presented as mean values ± standard deviation (SD) for age, or median values (25th–75th percentile) for disease duration. m: males; f: females; y: years; NS: non-significant. ^†^ χ^2^-test, ^‡^ 1-way ANOVA, ^§^ 2-way ANCOVA, *^a^* Bonferroni corrected *p* < 0.001 vs. ALS and 0.066 vs. FTD-ALS.

**Table 2:** Patients with *C9orf72* hexanucleotide repeat expansion.

Patient ID	Sex	Phenotype	Age at Onset	Age at Diagnosis	Family History	MRI	HMPAO-SPECT
1	F	FTD	61	63	Sister and 3 cousins ALS	Mild frontal atrophy and left sylvius and temporal pole.	NA
2	F	bvFTD	69	70	Mother with dementia	Mild frontal, temporal (L > R) and parietal atrophy	Frontal hypoperfusion (R > L) and right parietal
3	F	FTD-psychiatric symptoms	45	54	Mother FTD-ALS Maternal uncle ALS and aunt dementia	Mildfrontal atrophy and white matter lesions	Frontal hypoperfusion (L > R)
4	M	bvFTD	51	58	Mother ALS	Bilateral frontal strokes and frontal atrophy	NA
5	M	bvFTD with psychiatric symptoms	38	41	No	Frontal, temporal and parietal atrophy	NA
6	M	ALS	71	72	No	Mild global atrophy	NA
7	F	ALS	59	61	No	Frontal and parietal atrophy	NA
8	F	ALS	63	64	Sister ALS	NA	NA
9	F	ALS	42	43	Mother and 2 maternal aunts with ALS	NA	NA
10	M	FTD-ALS	48	50	Father with FTD	Diffuse atrophy, temporal > frontal	NA
11	F	FTD-ALS	43	44	Grandmother and 7/9 uncles with ALS; mother with dementia	Frontal, perisylvian atrophy (L > R), mild increase in signal intensity along the corticospinal tract	Diffuse frontal, temporal and parietal hypoperfusion
12	F	ALS	56	58	Mother with ALS; maternal cousin with ALS and *C9orf72* (+)	Mild ischemic microangiopathy	NA
13	M	FTD-ALS	44	45	Mother and 2 maternal uncles with motor disorder	Frontotemporal atrophy	NA (DATSCAN+)
14	F	ALS	41	43	Father with dementia; paternal aunt with ALS	Midline cerebellar dysplasia	NA

ALS: amyotrophic lateral sclerosis; FTD: frontotemporal dementia; bv: behavioral variant; NA: non-available.

**Table 3:** Patients with causative variants other than the *C9orf72* repeat expansion.

Patient ID	Sex	Phenotype	Age at Onset	Age at Diagnosis	Family History	Brain MRI	Gene	Transcript	Variant	gnomAD Frequency (%)	rs	CADD Score	MaxEnt-Scan (Splice Site Loss)
1	F	ALS (bulbar onset)	67	69	2 brothers; sister; father with ALS	Unremarkable	*TARDBP*	NM_007375.4	p.Met337Val (c.1009A>G)	≤0.001	80356730	22.4	-
2	F	ALS	63	64	No	Unremarkable			p.Asn352Ser (c.1055A>G)	0.000	80356734	18.6	-
3	M	FTD-ALS	57	60	No	Frontal, temporal atrophy			p.Ile383Val (c.1147A>G)	0.002	80356740	17.2	-
4	F	PPA	60.3	61	No	Frontal, temporal atrophy (L > R)	*GRN*	NM_002087.4	c.463-2A>G	0.000	-	33.0	From 3.77 to −4.19
5	M	PPA	50	60	Yes (grandmother)	Frontal, temporal, parietal atrophy (L > R)			c.934-1G>A	0.000	-	34.0	From 9.63 to 0.88
6	F	PPA	61	62	No	Perisylvian atrophy (L > R)			p.Cys482Tyr (c.1445G>A)	0.000	-	30.0	-
7	M	IBM/FTD	47	63	Yes (brother ALS; brother bvFTD)	Frontal lobe atrophy	*VCP*	NM_007126.5	p.Arg159His (c.476G>A)	≤0.001	121909335	23.2	-
8	M	IBM/FTD	58	68	Yes (mother ALS)	-							
9	M	IBM/FTD/PaD	43	53	No	-			p.Arg155His (c.464G>A)	0.000	121909329	24.6	-
10	M	ALS	79	81	No	Unremarkable	*FUS*	NM_001170634.1	p.Gly506Val (c.1517G>T)	0.000	-	23.7	-
11	M	ALS	36	37	Yes (brother ALS)	Unremarkable	*SOD1*	NM_000454.5	p.Ser106Leu (c.317C>T)	~0.000	1378590183	22.4	-

ALS: amyotrophic lateral sclerosis; FTD: frontotemporal dementia; IBM: inclusion body myositis; PaD: Paget’s disease.

## Data Availability

The data presented in this study are available on request from the corresponding author. The data are not publicly available due to privacy restrictions.
